# Supplementary material for: Unveiling the Bioactive Potential of the Invasive Jellyfish Phyllorhiza punctata Through Integrative Transcriptomic and Proteomic Analyses
Source: Biomolecules. 2025 Aug 4;15(8):1121. doi: 10.3390/biom15081121 (PMC12383608; doi:10.3390/biom15081121)
Supplement: Supplementary file 1 [file biomolecules-15-01121-s001.zip › Suplementary Material/FileS3/PEPFOLD4/XP_065060541_1625/clusters.html]

 PEP-FOLD clusters


Download archive of all models  here   

|  |  |  |
| --- | --- | --- |
| # | file | sOPEP |
| # cluster 1 - Size: 99 |  |  |
|  | XP\_065060541\_1625-model1 | -16.2453 |
|  | XP\_065060541\_1625-model1.1 | -16.1559 |
|  | XP\_065060541\_1625-model1.2 | -16.1479 |
|  | XP\_065060541\_1625-model1.3 | -16.1445 |
|  | XP\_065060541\_1625-model1.4 | -16.1275 |
|  | XP\_065060541\_1625-model1.5 | -16.1275 |
|  | XP\_065060541\_1625-model1.6 | -16.1275 |
|  | XP\_065060541\_1625-model1.7 | -16.1275 |
|  | XP\_065060541\_1625-model1.8 | -16.0953 |
|  | XP\_065060541\_1625-model1.9 | -16.0953 |
|  | XP\_065060541\_1625-model1.10 | -16.0953 |
|  | XP\_065060541\_1625-model1.11 | -16.0953 |
|  | XP\_065060541\_1625-model1.12 | -16.0953 |
|  | XP\_065060541\_1625-model1.13 | -16.0953 |
|  | XP\_065060541\_1625-model1.14 | -16.0953 |
|  | XP\_065060541\_1625-model1.15 | -16.0804 |
|  | XP\_065060541\_1625-model1.16 | -16.0804 |
|  | XP\_065060541\_1625-model1.17 | -16.0804 |
|  | XP\_065060541\_1625-model1.18 | -16.0577 |
|  | XP\_065060541\_1625-model1.19 | -16.0395 |
|  | XP\_065060541\_1625-model1.20 | -16.0338 |
|  | XP\_065060541\_1625-model1.21 | -16.0338 |
|  | XP\_065060541\_1625-model1.22 | -16.0315 |
|  | XP\_065060541\_1625-model1.23 | -16.0272 |
|  | XP\_065060541\_1625-model1.24 | -16.0272 |
|  | XP\_065060541\_1625-model1.25 | -16.0272 |
|  | XP\_065060541\_1625-model1.26 | -16.0272 |
|  | XP\_065060541\_1625-model1.27 | -16.0272 |
|  | XP\_065060541\_1625-model1.28 | -16.0272 |
|  | XP\_065060541\_1625-model1.29 | -16.0272 |
|  | XP\_065060541\_1625-model1.30 | -16.0272 |
|  | XP\_065060541\_1625-model1.31 | -16.0272 |
|  | XP\_065060541\_1625-model1.32 | -16.0272 |
|  | XP\_065060541\_1625-model1.33 | -16.0272 |
|  | XP\_065060541\_1625-model1.34 | -16.0125 |
|  | XP\_065060541\_1625-model1.35 | -16.0125 |
|  | XP\_065060541\_1625-model1.36 | -16.0125 |
|  | XP\_065060541\_1625-model1.37 | -16.0125 |
|  | XP\_065060541\_1625-model1.38 | -16.0125 |
|  | XP\_065060541\_1625-model1.39 | -16.0125 |
|  | XP\_065060541\_1625-model1.40 | -16.0125 |
|  | XP\_065060541\_1625-model1.41 | -16.0125 |
|  | XP\_065060541\_1625-model1.42 | -16.0125 |
|  | XP\_065060541\_1625-model1.43 | -16.0116 |
|  | XP\_065060541\_1625-model1.44 | -16.0116 |
|  | XP\_065060541\_1625-model1.45 | -16.0116 |
|  | XP\_065060541\_1625-model1.46 | -16.009 |
|  | XP\_065060541\_1625-model1.47 | -16.009 |
|  | XP\_065060541\_1625-model1.48 | -16.0055 |
|  | XP\_065060541\_1625-model1.49 | -16.0002 |
|  | XP\_065060541\_1625-model1.50 | -15.9836 |
|  | XP\_065060541\_1625-model1.51 | -15.9719 |
|  | XP\_065060541\_1625-model1.52 | -15.9719 |
|  | XP\_065060541\_1625-model1.53 | -15.9602 |
|  | XP\_065060541\_1625-model1.54 | -15.9602 |
|  | XP\_065060541\_1625-model1.55 | -15.9282 |
|  | XP\_065060541\_1625-model1.56 | -15.9282 |
|  | XP\_065060541\_1625-model1.57 | -15.9257 |
|  | XP\_065060541\_1625-model1.58 | -15.9257 |
|  | XP\_065060541\_1625-model1.59 | -15.9255 |
|  | XP\_065060541\_1625-model1.60 | -15.8922 |
|  | XP\_065060541\_1625-model1.61 | -15.8705 |
|  | XP\_065060541\_1625-model1.62 | -15.8677 |
|  | XP\_065060541\_1625-model1.63 | -15.8454 |
|  | XP\_065060541\_1625-model1.64 | -15.8454 |
|  | XP\_065060541\_1625-model1.65 | -15.8296 |
|  | XP\_065060541\_1625-model1.66 | -15.8296 |
|  | XP\_065060541\_1625-model1.67 | -15.8296 |
|  | XP\_065060541\_1625-model1.68 | -15.8296 |
|  | XP\_065060541\_1625-model1.69 | -15.8218 |
|  | XP\_065060541\_1625-model1.70 | -15.8094 |
|  | XP\_065060541\_1625-model1.71 | -15.8068 |
|  | XP\_065060541\_1625-model1.72 | -15.8068 |
|  | XP\_065060541\_1625-model1.73 | -15.7883 |
|  | XP\_065060541\_1625-model1.74 | -15.7463 |
|  | XP\_065060541\_1625-model1.75 | -15.7463 |
|  | XP\_065060541\_1625-model1.76 | -15.7386 |
|  | XP\_065060541\_1625-model1.77 | -15.729 |
|  | XP\_065060541\_1625-model1.78 | -15.6942 |
|  | XP\_065060541\_1625-model1.79 | -15.6942 |
|  | XP\_065060541\_1625-model1.80 | -15.6801 |
|  | XP\_065060541\_1625-model1.81 | -15.6801 |
|  | XP\_065060541\_1625-model1.82 | -15.665 |
|  | XP\_065060541\_1625-model1.83 | -15.6562 |
|  | XP\_065060541\_1625-model1.84 | -15.6562 |
|  | XP\_065060541\_1625-model1.85 | -15.6562 |
|  | XP\_065060541\_1625-model1.86 | -15.6463 |
|  | XP\_065060541\_1625-model1.87 | -15.6417 |
|  | XP\_065060541\_1625-model1.88 | -15.6067 |
|  | XP\_065060541\_1625-model1.89 | -15.5744 |
|  | XP\_065060541\_1625-model1.90 | -15.5728 |
|  | XP\_065060541\_1625-model1.91 | -15.5672 |
|  | XP\_065060541\_1625-model1.92 | -15.5578 |
|  | XP\_065060541\_1625-model1.93 | -15.4826 |
|  | XP\_065060541\_1625-model1.94 | -15.4759 |
|  | XP\_065060541\_1625-model1.95 | -15.4191 |
|  | XP\_065060541\_1625-model1.96 | -15.2613 |
|  | XP\_065060541\_1625-model1.97 | -15.2255 |
|  | XP\_065060541\_1625-model1.98 | -14.5211 |
| # cluster 2 - Size: 1 |  |  |
|  | XP\_065060541\_1625-model2 | -6.55869 |
|  |  |  |
